# Interlocking intramedullary nailing versus compression plating in the treatment of humeral fractures: Analysis of shoulder functional recovery, complication rates, and risk factors for reoperation

**DOI:** 10.12669/pjms.42.9.14108

**Published:** 2026-09

**Authors:** Dingtao Li, Zhanshan Zhang, Qiang Wang

**Affiliations:** 1 Dingtao Li, Department of Orthopedics, Baoding Forensic Hospital, Baoding 071000, Hebei, China; 2 Zhanshan Zhang Department of Orthopedics, Baoding Jingxiu District Hospital, Baoding 071000, Hebei, China; 3 Qiang Wang Orthopedics and Traumatology, Xushui District Hospital of Traditional Chinese Medicine, Baoding 071000, Hebei, China

**Keywords:** Complication, Compression plating, Humeral fracture, Interlocking intramedullary nailing, Risk factor for reoperation, Shoulder function

## Abstract

**Objective:**

To explore the effects and reoperation risks of interlocking intramedullary nailing(IMN) versus compression plating for the treatment of humeral fractures.

**Methodology:**

This was a retrospective study. Sixty patients with humeral fractures admitted to Baoding Forensic Hospital between November 2022 to November 2024 were enrolled as the study subjects. Information about surgical parameters (duration of operation [DoO], intraoperative blood loss[IBL]), postoperative shoulder function recovery, complications rates, and risk factors for reoperation were collected. Patients were divided into two major groups, receiving interlocking IMN (observation cohort, n=30) and compression plating (control cohort, n=30). t-test and chi-square test are used to compare the differences between two groups..

**Results:**

The observation group had a shorter DoO and less IBL than the control group (P<0.05). The observation group showed faster recovery of shoulder joint function at three and six months after surgery than control cohort (P<0.05). Complications rates in the observation group was lower than in the control cohort (P<0.05). Results showed that there were statistically significant differences in DoO, fracture type, osteoporosis, surgical approach, and postoperative complications between the non-reoperation group and the reoperation group (P<0.05).

**Conclusion:**

After internal fixation with interlocking intramedullary nail for humeral fracture, shoulder joint function recovers faster, operation related indicators are better, complication rate and reoperation risk factors are lower.

## INTRODUCTION

Humeral fractures are among the more common types of upper limb fractures, particularly resulting from traumatic events such as traffic accidents, sports injuries, and falls.^1^ A variety of treatment methods are available for humeral fractures, including conservative and surgical approaches. In recent years, with advancements in internal fixation techniques, surgical treatment has become a pivotal method for managing humeral fractures. The main internal fixation techniques include compression plating and interlocking intramedullary nailing (IMN). Compression plating stabilizes the fracture using plates and screws, creating a mechanically stable environment to facilitate fracture healing.^2^ Interlocking IMN, as a minimally invasive procedure, stabilizes fracture ends through the combined fixation of intramedullary nails and locking screws. This approach is characterized by minimal surgical trauma and reliable fixation. Due to its minimally invasive nature and favorable biomechanical properties, interlocking IMN has garnered significant attention.^3^

On the other hand, compression plating is widely used for its stable fixation outcomes. However, the comparative outcomes of these two techniques in terms of shoulder functional recovery, complication rates, and reoperation risks remain inconclusive. Shoulder functional recovery is a critical metric for evaluating the treatment outcomes of humeral fractures, as it directly impacts patients’ daily activities and work capabilities. Additionally, complications and reoperation risks are closely related to patient safety and the long-term prognosis. This study aimed to provide clinical insights into the treatment of humeral fractures by conducting a comparative analysis of shoulder functional recovery, complication rates, and risk factors for reoperation between interlocking IMN and compression plating.

## METHODOLOGY

This was retrospective research. A total of sixty patients suffering from humeral fractures, who were treated at Baoding Forensic Hospital from November 2022 to November 2024, were assigned to two cohorts according to different treatment methods: the interlocking intramedullary nailing (IMN) cohort (observation cohort) and the compression plating cohort (control cohort), with each cohort consisting of thirty patients.

### Ethical approval:

The study was approved by the Institutional Ethics Committee of Baoding Forensic Hospital (No.:2024-05-0001; Approval Date: May 13, 2024), and written informed consent was obtained from all participants.

### Inclusion criteria:

- Adults aged 18 years and older.
- Patients with fresh, closed humeral fractures.
- Patients with humeral fractures requiring surgical treatment as determined by clinical evaluation and imaging findings.
- All patients provided signed informed consent, agreeing to participate in the study and undergo subsequent follow-up evaluations.

### Exclusion criteria:

- Patients with pathological fractures.
- Patients with Any fracture of coracoid, calcification of CC ligament, osteolysis of acromion.
- Patients with a prior history of shoulder or upper limb surgeries.
- Patients with severe cardiovascular diseases, poorly controlled diabetes, or other serious systemic diseases.
- Patients with psychiatric disorders that prevent cooperation with treatment and follow-up evaluations.

The study’s observation cohort included 17 males and 13 females, ranging in age from 23 to 66 years, with a mean age of (44.2 ± 10.8) years. Regarding fracture types, 15 participants were diagnosed with humeral shaft fractures, while the remaining 15 had proximal humeral fractures. The interval between injury and surgical intervention varied from one to five days, averaging (2.5 ± 1.2) days. Conversely, the control cohort consisted of 19 males and 11 females, aged between 21 and 69 years, with a mean age of (46.5 ± 11.3) years. The study included a total of 30 cases of humeral fractures, comprising of 14 cases of humeral shaft fractures and 16 cases of proximal humeral fractures. The duration from the occurrence of the injury to the surgical intervention varied from one to six days, with an average time of (2.8 ± 1.5) days. No significant disparities were observed between the two cohorts in terms of gender, age, fracture type, or the period between injury and surgery (all P > 0.05).

### Surgical approaches:

The observation group underwent interlocking IMN: The individual was positioned supine while under general anesthesia, with the injured limb abducted at a 90° angle and the shoulder and elbow slightly flexed. A longitudinal incision measuring roughly 3-5 cm was created on the lateral aspect of the acromion, followed by a careful layer-by-layer dissection to access the fascia. The deltoid muscle was bluntly separated to fully expose the proximal humeral medullary canal entry point. Using a nail guide, the canal was accessed starting from the distal humerus and gradually advanced through the medullary cavity. Radiographic imaging was used to confirm the nail guide position, ensuring accurate alignment of the nail path without deviation. The intramedullary nail was locked in place, and locking screws were inserted sequentially to stabilize the nail on both ends of the fracture. The length (average: 18.5 ± 2.5 cm) and diameter (average: 9.2 ± 1.2 mm) of the locked intramedullary nail were selected based on the fracture type. After inserting the nail into the medullary canal, proximal and distal locking screws were installed. Fluoroscopic imaging was performed again to confirm proper fracture reduction and fixation positioning. The surgical site was irrigated with normal saline, and the wound was closed layer by layer. The average duration of operation (DoO) was (82.3 ± 14.5) minutes, and the average intraoperative blood loss (IBL) was (95.6 ± 23.4) mL.

The control group received internal fixation using a compression plate: The patient was placed in a supine position under general anesthesia, with the shoulder on the affected side slightly elevated. Using the fracture site as the center, a surgical incision of approximately 8-12 cm was made along the anterolateral or lateral aspect of the humerus, dissecting layer by layer to the fascia. The muscles were bluntly separated to expose the fracture ends. Hematoma and embedded tissue were carefully removed, and the fracture was reduced. A compression plate appropriate for the humeral anatomy (average length: 12.5 ± 3.0 cm) was selected and placed at the corresponding site of the humerus. Holes were drilled using an electric drill, and screws were inserted to secure the plate. Fluoroscopic imaging with a C-arm X-ray machine was used to confirm satisfactory fracture reduction and fixation. A drainage tube was placed before closing the wound. The average DoO was (110.2 ± 22.1) minutes, and the average IBL was (160.5 ± 35.2) mL.

All patients were treated with interlocking IMN and compression plate by strictly trained personnel, and the researchers were proficient in both fixation methods.Postoperative management was the same between the two groups. Both groups received standard antibiotic prophylaxis to prevent infection for 3-5 days postoperative. The affected limb was immobilized based on the specific fracture condition. The drainage tube was removed 24-48 hours postoperative. Starting from the first postoperative day, patients were guided to perform active flexion and extension exercises of the fingers and wrist joints. From weeks 2-3 postoperative, elbow joint flexion and extension exercises were initiated, with a range of motion gradually increasing from 0-30° to 0-90°. At 4-6 weeks postoperative, depending on fracture healing progress, passive abduction and forward flexion movements of the shoulder joint were gradually performed with the assistance of a rehabilitation therapist, beginning with a 30° range of motion and increasing by approximately 15° per week. Between 8-12 weeks postoperative, active shoulder joint exercises and strength training were gradually introduced, such as resistance exercises using elastic bands.

### Comparison of outcome measures:

Surgical Parameters: Detailed records were kept for the DoO and IBL for both groups.

### Shoulder functional recovery:

The Constant-Murley Scoring System was used to evaluate shoulder function. Assessments were conducted before surgery and at three and six months postoperative. The assessment consists of four subscales, amounting to a maximum of 100 points:

- Pain, which accounts for 15 points;
- Activities of Daily Living (ADLs), worth 20 points;
- Range of Motion (ROM) covering shoulder flexion, internal rotation, external rotation, and abduction, contributing 40 points; and Strength, totaling 25 points. The overall score ranges up to hundred points, with higher scores reflecting improved recovery of shoulder function. Additionally, the incidence of postoperative complications, such as fixation loosening, nonunion of fractures, nerve injuries, and infections, was meticulously tracked and documented.
- Univariate Analysis of Reoperation vs. Non-Reoperation Groups: Those patients who had a subsequent surgery were categorized into the reoperation group, while the remaining patients were placed in the non-reoperation group. The two groups were compared based on various factors, including sex, age, date of operation (DoO), type of fracture, presence of osteoporosis, surgical method, and postoperative complications.
- Identifying Risk Factors for Reoperation: Patient information was gathered, encompassing DoO, osteoporosis status, type of fracture, surgical technique, and postoperative complications. A multivariate logistic regression analysis was performed to identify independent risk factors associated with reoperation.

### Statistical analysis:

Data analysis was conducted utilizing the statistical software SPSS 25.0. For measurement data, the Kolmogorov Smirnov test is first used to test whether the data follows a normal distribution., if the measurement data were presented as mean ± standard deviation (*x̅*±*s*), comparisons were performed using the t-test., and t-test is used for inter group comparison; Otherwise, median (interquartile range) will be used for statistical description, and non-parametric tests will be used for inter group comparison. The categorical data were expressed as Frequency (percentages),comparisons were carried out using the χ² test. Results were considered significant at p<0.05.

## RESULTS

DoO in the observation cohort was meaningfully shorter than in the control cohort (*P <* 0.05). Additionally, IBL in the observation cohort was meaningfully less than in the control cohort (*P <* 0.05). Table-I.

**Table-I:** Comparison of surgical parameters.

Group	n	DoO (min)	IBL (mL)
Observation group	30	82.3±14.5	95.6±23.4
Control group	30	110.2±22.1	160.5±35.2
*t-value*		5.781	8.410
*P-value*		<0.001	<0.001

Before surgery, the Constant-Murley score (CMS) in the observation cohort was higher than that in the control cohort, but the contrast lacked statistical significance (*P >* 0.05). At three and six months postoperative, the CMS in the observation cohort was meaningfully higher than in the control cohort (*P <* 0.05, respectively). Table-II.

**Table-II:** Comparison of shoulder function recovery levels based on CMS.

Group	n	Preoperative	Three months postoperative	Six months postoperative
Observation group	30	46.2±8.8	68.5±9.2	82.1±11.3
Control group	30	45.5±9.0	60.2±10.5	73.5±12.8
*t-value*		0.305	3.256	2.759
*P-value*		0.762	0.002	0.008

The observation cohort reported three cases of complications, while the control cohort had 10 cases. The complication rate in the observation cohort was meaningfully lower than that in the control cohort (*P <* 0.05). Table-III.

**Table-III:** Comparison of complication incidence.

Group	n	Fixation loosening	Fracture nonunion	Nerve injury	Infection	Complication rate
Observation group	30	1	1	1	0	3(10.00)
Control group	30	3	3	2	2	10(30.00)
*χ²*-value		-	-	-	-	4.812
*P*-value		-	-	-	-	0.028

Patients who underwent reoperation after surgery were assigned to the reoperation cohort (*n* = 6), while those without reoperation formed the non-reoperation cohort (*n* = 54). Univariate analysis showed that the two cohorts showed statistically meaningful contrasts in DoO, fracture type, osteoporosis, surgical approach, and postoperative complications (*P <* 0.05). No meaningful contrasts were found in other factors between the two cohorts (P > 0.05). Table-IV.

**Table-IV:** Univariate analysis of differences between the reoperation and non-reoperation groups.

Factor	Non-reoperation group (n = 54)	Reoperation group (n = 6)	χ²	P-value
Sex (n)			0.008	0.930
Male	33	3		
Female	21	3		
Age (n)			0.031	0.860
≥60 y/o	21	2		
<60 y/o	33	4		
DoO	89.23±8.5	97.31±8.3	2.213	0.031
Fracture type			4.271	0.039
Humerus shaft fracture	29	0		
Proximal humeral fracture	25	6		
Osteoporosis			4.565	0.033
Yes	10	4		
No	44	2		
Surgical approach			4.630	0.031
Interlocking IMN	30	0		
Compression plating	24	6		
Postoperative complications			5.281	0.023
Present	9	4		
Absent	45	2		

A binary logistic regression analysis was conducted with “reoperation (“0” was assigned for “Yes” and “1” for “No”)” as the dependent variable, and “DoO, osteoporosis, fracture type, surgical approach, and postoperative complications” as independent variables. The analysis showed that osteoporosis, fracture type, surgical approach, and postoperative complications were significant risk factors for reoperation in patients with humeral fractures(*P<* 0.05), Table-V.

**Table-V:** Multivariate logistic regression analysis of reoperation risk factors.

Risk factor	β	S.E.	Wald	df	P-value	OR	95% CI	
							Minimum value	Maximum value
DoO	1.698	1.107	2.353	1	0.125	0.183	0.021	1.602
Osteoporosis	2.034	0.760	7.169	1	0.007	0.131	0.030	0.580
Fracture type	1.544	0.698	4.901	1	0.027	4.685	1.194	18.386
Surgical approach	1.892	0.959	3.893	1	0.048	0.151	0.023	0.987
Postoperative complications	0.144	0.060	5.678	1	0.017	1.155	1.026	1.229
Constant	0.142	0.071	3.982	1	0.046	1.153	1.000	1.326

## DISCUSSION

This study involved 60 patients with humeral fractures who were treated with either interlocking IMN or compression plating. A comparative analysis was conducted to examine differences in shoulder functional recovery, complication rates, and reoperation risk factors, yielding a series of meaningful results. The following is an in-depth discussion of these findings. Regarding surgical parameters, DoO in the observation group was significantly shorter than in the control group, and IBL was also significantly reduced. This is primarily due to the more direct and minimally invasive surgical approach, interlocking IMN.^4^ The procedure involves inserting a guidewire through a small incision at the apex of the greater tuberosity and placing the intramedullary nail, without the need for extensive periosteal dissection and soft tissue mobilization to expose the fracture site and position the plate, as required in compression plating.^5^,^6^ This smaller surgical incision not only reduced the DoO but also lowered the risk of bleeding due to tissue damage.^7^

Regarding shoulder functional recovery, the CMS at different postoperative time points showed that the observation group outperformed the control group. From a biomechanical perspective, interlocking IMN is positioned within the humeral medullary canal, which is closer to the humerus’s mechanical axis. This allows for a more accurate simulation of the normal stress pattern of the humerus during the fracture healing process.^8^ As a result, the fracture site heals within a relatively stable mechanical environment, facilitating the coordinated recovery of surrounding muscles, tendons, and other soft tissues of the shoulder joint.^9^ In contrast, compression plating is applied to the lateral or anterolateral aspect of the humerus, altering the original stress distribution of the bone. This can lead to stress shielding under the plate, causing osteoporosis, which may subsequently affect the long-term function of the shoulder.^10^ Additionally, the larger surgical trauma associated with compression plating can lead to adhesions and scar tissue formation in the shoulder, restricting its ROM.

In terms of complication rates, the observation group had a dramatically lower incidence compared to the control cohort. The design of the nail allows it to evenly distribute stress while fixing the fracture site, reducing the likelihood of internal fixation loosening.^11^ Additionally, its minimal disruption to the blood supply of the fracture site preserves more periosteum and surrounding soft tissue circulation, providing a favorable biological environment for fracture healing,^12^ which lowers the risk of nonunion. Furthermore, the smaller surgical incision reduces the risk of postoperative infection, and the relatively gentle manipulation of surrounding tissues, such as nerves, during surgery also lowers the risk of nerve damage.^13^,^14^ In contrast, compression plating, due to the stress shielding effect, reduces the stress beneath the plate, leading to abnormal bone remodeling. This increases the likelihood of fixation loosening and fracture nonunion. Moreover, the larger surgical incision increases the risk of infection^15^, and the extensive dissection and traction of surrounding tissues during the procedure raise the probability of nerve damage.^16^ In terms of risk factors for reoperation, single factors such as surgery time, osteoporosis, fracture type, surgical method, and postoperative complications have a significant impact on the risk of reoperation.

Regarding reoperation risk factors, the multivariate logistic regression analysis identified several significant factors, including DoO, osteoporosis, fracture type, surgical approach, and postoperative complications, all of which have a substantial impact on the risk of reoperation. Among fracture types, comminuted fractures and other complex fractures, characterized by multiple fracture fragments and irregular fracture lines, present significant challenges for reduction and fixation no matter which surgical approach was adopted. For these types of fractures, the choice of surgical approach is particularly crucial.^17^ If the selected internal fixation technique does not provide adequate stability or causes excessive disruption to the blood supply at the fracture site, it is more likely to result in poor fracture healing, thus increasing the risk of reoperation.^18^ In this study, the observation group overall performed better regarding these reoperation risk factors, further confirming its reliability and superiority in treating humeral fractures.

However, in clinical practice, we should not solely rely on the results of this study to unilaterally choose interlocking IMN over compression plating. While interlocking IMN demonstrates advantages in many aspects, there are specific situations where compression plating may still be the more appropriate choice. For elderly patients with proximal humeral fractures involving the joint surface and severe osteoporosis, compression plates can provide better support for the joint surface and stronger fixation strength. This approach also allows for early functional rehabilitation, reducing the risk of complications such as shoulder stiffness.^19^ On the other hand, for simple humeral shaft fractures in younger patients, interlocking IMN can fully utilize their minimally invasive and quick recovery advantages. Moreover, the experience and technical proficiency of the surgeon also significantly influence the outcome of the surgery and the incidence of complications. Whether using interlocking IMN or compression plating, the surgeon must be proficient in surgical techniques and strictly adhere to operational guidelines to ensure the success of the surgery and the patient’s favorable prognosis.^20^

### Limitations:

This study is limited by a small sample size.

## CONCLUSIONS

The results of this study indicate that interlocking IMN is superior to compression plating in several aspects for treating humeral fractures. However, in clinical practice, it is still essential to comprehensively consider individual differences, fracture type, and the surgeon’s experience when selecting the most appropriate treatment plan. At the same time, postoperative rehabilitation and long-term follow-up are crucial elements for improving treatment outcomes, reducing complication rates, and minimizing reoperation risks, and they require sufficient attention.

### Recommendations:

Future studies could further expand the sample size and conduct more detailed stratified analyses of different types of humeral fractures to better define the optimal indications for both internal fixation techniques, providing more precise and scientifically grounded guidance for the treatment of humeral fractures.

### Authors’ Contributions:

**DL:** Designed this study and prepared this manuscript, and is responsible and accountable for the accuracy or integrity of the work in authors’ contribution.

**ZZ:** Collected and analyzed clinical data. Critical Review

**QW:** Participated in acquisition, analysis, or interpretation of data and prepared the manuscript.

All authors have read and approved the final manuscript.
